# Transient increase of activated regulatory T cells early after kidney transplantation

**DOI:** 10.1038/s41598-018-37218-x

**Published:** 2019-01-31

**Authors:** Young-Seon Mederacke, Florian W. Vondran, Sonja Kollrich, Elvira Schulde, Roland Schmitt, Michael P. Manns, Jürgen Klempnauer, Reinhard Schwinzer, Fatih Noyan, Elmar Jaeckel

**Affiliations:** 1 Department of Gastroenterology, Hepatology & Endocrinology, Hannover Medical School, Hannover, Germany; 2 Department of General, Visceral and Transplantation Surgery, Hannover Medical School, Hannover, Germany; 3 Department of Nephrology, Hannover Medical School, Hannover, Germany; 4 Integrated Research and Treatment Center, Transplantation (IFB-Tx), Hannover Medical School, Hannover, Germany

## Abstract

Regulatory T cells (Tregs) are crucial in controlling allospecific immune responses. However, studies in human kidney recipients regarding the contribution of polyspecific Tregs have provided differing results and studies on alloreactive Tregs are missing completely. In this retrospective study, we specifically analyzed activated CD4^+^CD25^high^FOXP3^+^GARP^+^ Tregs in 17 patients of a living donor kidney transplantation cohort longitudinally over 24 months by flow cytometry (FOXP3: forkhead box protein 3, GARP: glycoprotein A repetitions predominant). We could demonstrate that Tregs of patients with end-stage renal disease (ESRD) are already pre-activated when compared to healthy controls. Furthermore, even though total CD4^+^CD25^high^FOXP3^+^ Treg numbers decreased in the first three months after transplantation, frequency of activated Tregs increased significantly representing up to 40% of all peripheral Tregs. In a cohort of living donor kidney transplantation recipients with stable graft function, frequencies of activated Tregs did not correlate with the occurrence of acute cellular rejection or chronic graft dysfunction. Our results will be important for clinical trials using adoptive Treg therapy after kidney transplantation. Adoptively transferred Tregs could be important to compensate the Treg loss at month 3, while they have to compete within the Treg niche with a large number of activated Tregs.

## Introduction

Regulatory T cells (Tregs) play a pivotal role in immune regulation mediating self-tolerance and tolerance to alloantigens by suppressing effector T cells^1^. In murine transplant models, polyspecific CD4^+^CD25^high^FOXP3^+^ Tregs have been proven to be effective in controlling an allogeneic T cell response under lymphopenic conditions^2^, whereas under non-lymphopenic conditions polyspecific Tregs were not sufficient to prevent allograft rejection^3,4^. Yet, several murine studies have demonstrated, that immunosuppressive capacities of Tregs could be markedly improved by the use of antigenspecific instead of polyspecific Tregs^5–10^.

Although murine data clearly suggest a major role of Tregs in allogeneic tolerance, studies in human organ recipients have been less clear and partly contradictory. Especially kidney transplant recipients have been investigated intensively: quantitative FOXP3 mRNA analysis linked elevated intragraft FOXP3 levels not only with acute cellular rejection (ACR)^11–13^, but also subclinical rejection^14,15^ and borderline changes^16,17^. Others reported comparable FOXP3 mRNA levels in tolerant and non-tolerant patients^18^. Studies on circulating Tregs displayed lower numbers of CD4^+^CD25^high^FOXP3^+^ Tregs in chronic rejection, whereas kidney recipients with stable allograft function and operational tolerance had similar Treg frequencies compared to healthy controls^19–22^. However, all of these studies failed to demonstrate superior immunosuppressive potencies of Tregs of tolerant patients after polyclonal stimulation. Game *et al*. even postulated in 2003, that CD4^+^CD25^+^ Tregs are not significantly involved in donor-specific hyporesponsiveness in patients with stable allograft function^20^, which was partly contradicted by Velthuis *et al*. in 2006^22^.

None of the studies focused on allospecificity and activation status of Tregs, as a specific marker for the detection of alloactivated Tregs had been missing. We and others have identified glycoprotein A repetitions predominant (GARP) as reliable marker for the detection of activated Tregs^6,23^. Therefore, we aimed to specifically analyze the donor-specific Treg activation after kidney transplantation in a living donor kidney transplantation cohort. We aimed to further answer several questions: (i) Does frequency of donor-reactive Treg change after allogeneic kidney transplantation? Do Tregs of tolerant patients display donor-specific hyporesponsiveness or a higher activation level? (ii) Do alloreactive Tregs expand after transplantation? And (iii) does Treg activation correlate with allograft tolerance, function or histological changes?

To our knowledge, our study is the first, analyzing the impact of the allogeneic immune response on the activation level in Tregs after kidney transplantation.

## Results

### Variable frequency of alloreactive Tregs in healthy subjects after stimulation with different allogeneic stimuli

**Figure 1:**
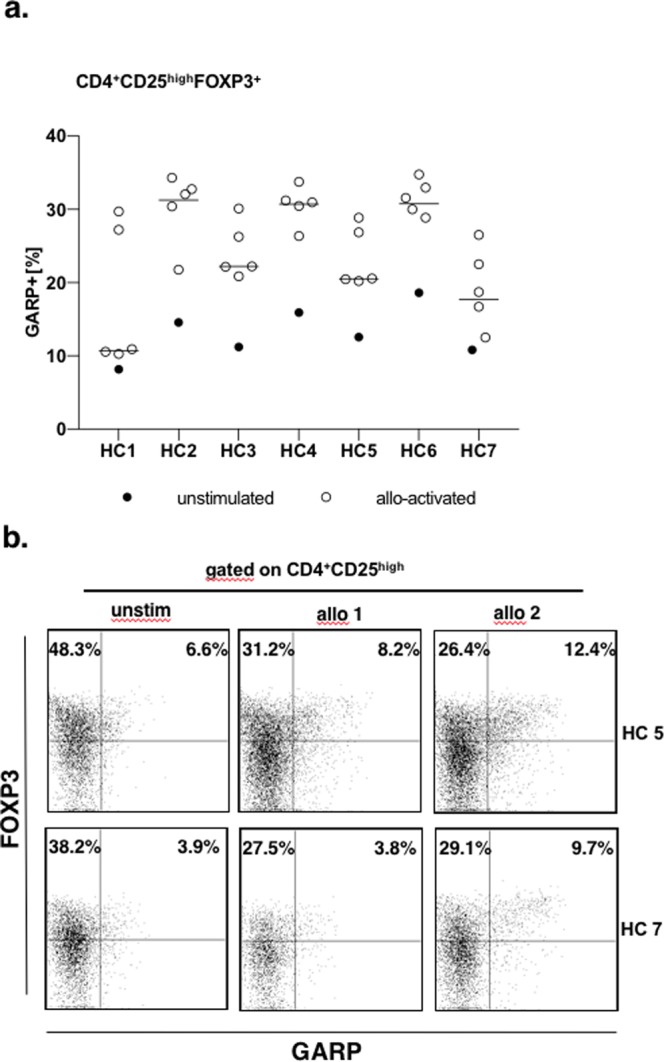
Frequency of alloreactive Tregs after allogeneic stimulation. (**a**) PBMC of seven healthy volunteers (HC1–HC7) were stimulated with PBMC of five different PBMC donors. Background activation was determined in unstimulated PBMC of each healthy volunteer (unstimulated, black dots). Donor PBMCs were identified by CFSE positive staining and further excluded. Recipient’s PBMC were gated on CFSE^-^CD4^+^CD25^high^ T cells. Allogeneically activated Tregs were further identified by their expression of FOXP3 and GARP (for detailed gating strategy see Supplementary Fig. 1). Frequency of allogeneically activated Tregs was expressed as percentage of all Tregs by calculating the ratio of CD4^+^CD25^high^FOXP3^+^GARP^+^ (activated Tregs) to CD4^+^CD25^high^FOXP3^+^ (total Tregs). (**b**) Representative dot plots of two different healthy individuals (HC5 and HC7) after allogeneic stimulation, populations are gated on CD4^+^CD25^high^ T cells. Activated Tregs are defined by their co-expression of FOXP3 and GARP (upper right quadrant). Left column shows unstimulated PBMC, middle and right panel show activated Tregs after allogeneic stimulation with two different allogeneic stimuli.

Up to 10% of the T cell repertoire are considered to be alloreactive^24^. We analyzed the precursor frequencies of alloreactive Tregs in seven healthy volunteers, each being stimulated with 5 different allogeneic PBMC (peripheral blood mononuclear cells) donors (Fig. 1). In unstimulated PBMCs background precursor frequencies of activated Tregs ranged from 8.1% to 18.6% (13.1 ± 3.5%). After 24 hours *in vitro* stimulation with allogeneic PBMC the frequency of activated Tregs increased up to 34.8% (25.3 ± 1.2%, range 10.2–34.8%). Notably, in the same subject frequencies of alloreactive Tregs varied depending on the deployed allogeneic stimulus, resulting in an up to threefold stronger alloactivation in Tregs of the same individual to a different allogeneic stimulus.

### Increased number of activated Tregs in patients on chronic hemodialysis

Several studies have been performed questioning frequency and function of Tregs in patients with ESRD. So far, results have been inconsistent: Increased, similar as well as decreased Treg frequencies in patients with ESRD have been reported^25–29^. We also analyzed the frequency of regulatory Tregs in patients with ESRD on chronic hemodialysis. As Treg frequencies in pre-transplantation (pre-Tx) samples of the transplant group were comparable to samples from the HD-group (Supplementary Fig. 2), both groups were combined for analysis as ESRD group (pre-Tx + HD). Two patients from the transplant group were excluded, as they did not receive hemodialysis prior to transplantation (one patient performed peritoneal dialysis, the other was transplanted preemptively).

**Figure 2:**
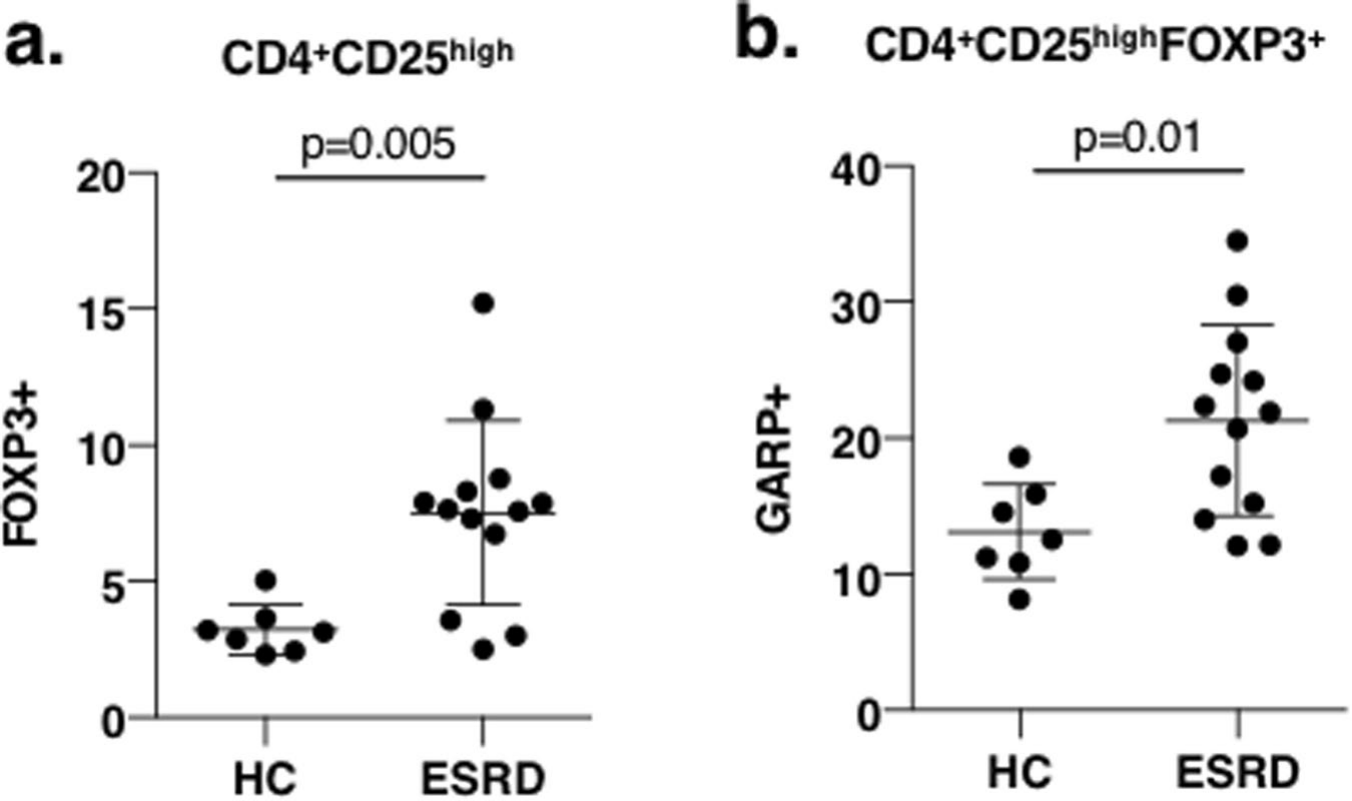
Increased Treg frequency and activation in patients with end-stage renal disease. (**a**) Frequency of CD4^+^CD25^high^ FOXP3^+^ polyspecific regulatory T cells was assessed in 13 patients with ESRD and seven healthy controls (HC) by flow cytometry. Frequency of polyspecific Tregs was significantly increased in ESRD patients (HC 3.2 ± 0,9% vs. ESRD 7.5 ± 3.4%, p = 0.005). (**b**) Frequency of endogenously activated Tregs in ESRD patients was compared to healthy controls. Activated Tregs were identified as CD4^+^CD25^high^FOXP3^+^GARP^+^. Percentage of activated Tregs of all Tregs was calculated by the ratio of GARP^+^ Tregs of all Tregs. Patients with ESDR displayed a significantly higher activation status of Tregs than healthy controls (HC 13.1 ± 0,3.5 vs. HD 21.3 ± 7.0%, p = 0.01). Data are presented as mean ± SD.

In contrast to the majority of the studies mentioned above, we observed significantly increased frequencies of polyspecific Tregs in ESRD (Fig. 2a; HC 3.2 ± 0.9% vs. ESRD 7.5 ± 3.4%, p = 0.0045). More importantly, also Treg activation level in patients on hemodialysis was markedly increased in comparison to healthy controls (Fig. 2b; HC 13.1 ± 0.3.5 vs. HD 21.3 ± 7.0%, p = 0.013).

### Determination of specificity of activated Tregs

**Figure 3:**
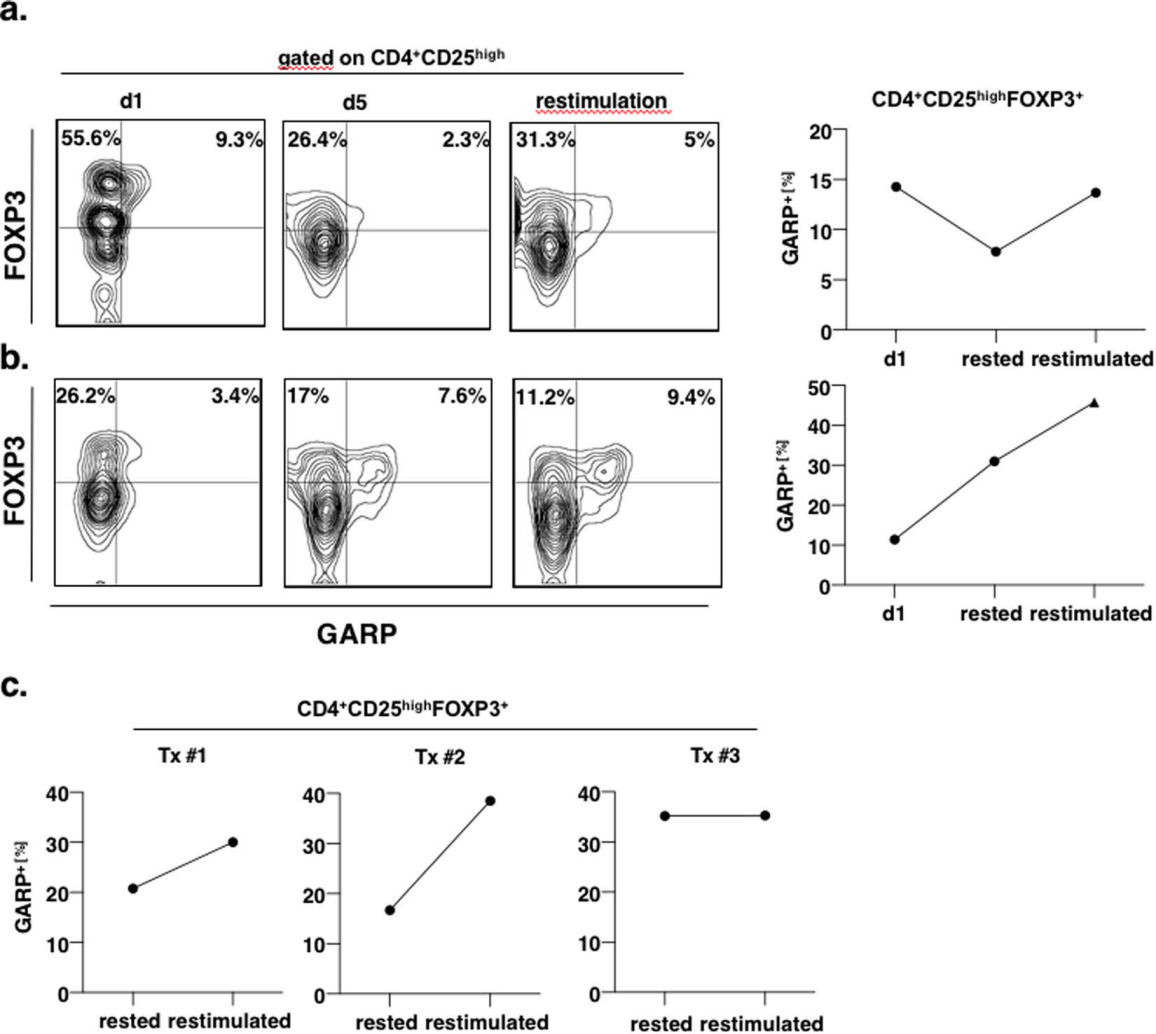
Specificity of activated Tregs. (**a**,**b)** Two representative recall experiments are shown. After thawing, PBMC of kidney recipients were rested overnight in culture medium before expression of GARP in Tregs was analyzed by flow cytometry the next day (d1, left column). After five days rest samples were reanalyzed for downregulation of GARP expression (d5, middle column). At day 5 Tregs were restimulated with donor-antigen (restimulation, right column). (**a**) Represents an example of sufficient downregulation of GARP to baseline levels and adequate increase of activation marker GARP as donor-specific immune response. (**b**) Represents an example of insufficient downregulation and strong expression of activation marker GARP, which is independent from the restimulation with donor-antigen. T cells were gated on CD4^+^CD25^high^, allogeneically activated Tregs were identified by their co-expression of FOXP3 and GARP. Frequency of allogeneically activated Tregs was expressed as percentage of all Tregs in analogy to Fig. 1. (**c**) Three representative analysis of allogeneic activation of Tregs from renal transplant recipients. The majority of the samples was susceptible to allogeneic stimulation after after five day rest in culture medium as described in section 3A (left and middle plot), whereas few samples remained unresponsive to further stimulation (right plot).

As Tregs of patients with ESRD already present an activated phenotype, direct analysis of Tregs does not allow conclusions on antigen specificity of Tregs. In naive Tregs GARP is up-regulated within 24 h after T cell receptor activation and GARP expression returns to normal within 96 h without any further Treg stimulation^6^. Therefore, we rested Tregs before stimulation with donor material. First, isolated PBMC from renal transplant recipients were rested in culture medium to achieve downregulation of GARP on Tregs to baseline levels before undergoing further allogeneic stimulation. After five days’ rest, PBMC were re-stimulated with their corresponding donor MHC, resulting in an upregulation of GARP expression in alloreactive Tregs (Fig. 3a,c left and middle plot). In part of the samples, resting of Tregs did not lead to downregulation of GARP. In these cases, Tregs remained unresponsive to further allogeneic stimulation (Fig. 3c right plot) or showed a donor-unspecific Treg activation pattern (Fig. 3b).

### Activated Tregs increase after kidney transplantation

**Figure 4:**
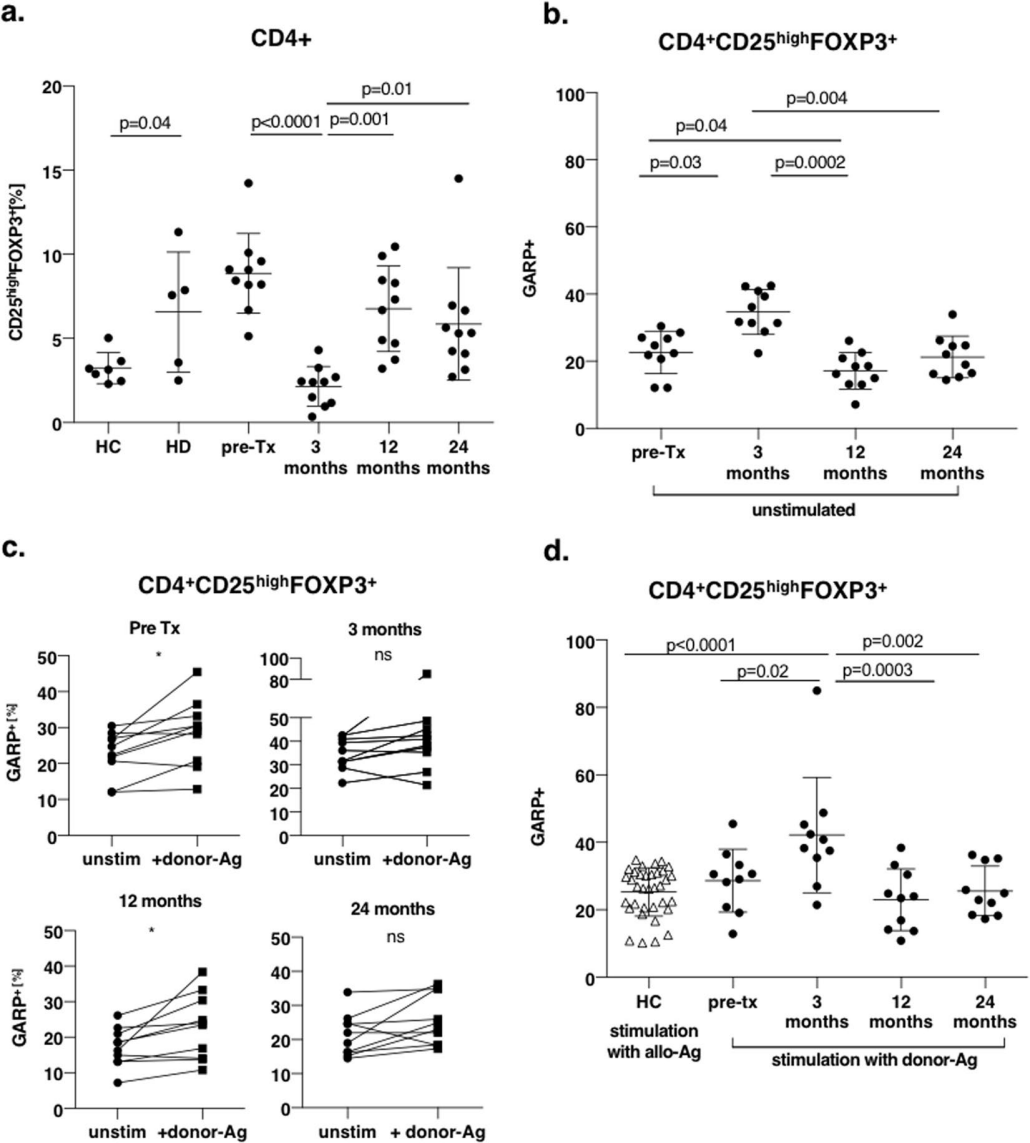
Activated regulatory T cell increase after kidney transplantation. PBMCs of renal transplant recipients collected pre-Tx and at month 3, 12, and 24 post-Tx were stimulated with their corresponding donor PBMC for 24 hours *in vitro* and subsequently stained for CD4, CD25, FOXP3 and GARP. (**a**) Percentage of CD4^+^CD25^high^FOXP3^+^ polyspecific regulatory T cells dropped significantly from pre-Tx to 3 months after transplantation (8.9 ± 2.4% vs. 2.2 ± 1.8%, p < 0.0001) and recovered until 12 months post-Tx (6.8 ± 2.6%, p = 0.001). Healthy controls (HC) and patients on chronic hemodialysis (HD) served as controls. (**b**) Frequency of endogenously activated regulatory T cells was determined in unstimulated PBMC samples. After thawing, PBMC were rested for 5 days before analysis by flow cytometry was performed. Percentage of GARP^+^ Tregs of total CD4^+^CD25^high^FOXP3^+^ regulatory T cells was calculated. Data are presented as mean ± SD. (**c**) After *in vitro* stimulation with donor-Ag increase of frequency of activated Tregs was noted in the early post-Tx course (pre-Tx 28.7 ± 9.3% vs 3 months 42.2 ± 17.1%, p = 0.043) and declined to pre-Tx levels until 12 months post-Tx (23 ± 9.1%, p = 0.006). From 12 to 24 months activation level of Tregs stayed stable (25.6 ± 7.3%, p = 0.66). (**d**) Baseline frequency of activated Tregs in unstimulated, rested samples (unstim) and after allogeneic stimulation with donor PBMC (+donor-Ag) in the longitudinal post-Tx course.

Following renal transplantation, the recipient’s immune system is constantly challenged by the allogeneic graft. As we were interested in how the Treg compartement changes in the setting of renal transplantation, we performed a longitudinal analysis of Treg frequency in ten kidney transplant recipients. Of these ten patients enrolled in the longitudinal analysis, eight patients had a stable post-transplantation (post-Tx) course, whereas two patients had experienced episodes of acute cellular rejection at day 7 post-Tx (ACR patient 1) and at day 40 and 90 post-Tx (ACR patient 2). Previously others reported that Treg frequencies decline in the initial time after transplantation^30–33^, which we also confirmed in our cohort. Three months after transplantation we observed a significant drop in polyspecific Treg frequencies in peripheral blood by 4-fold (Supplementary Fig. 4a), that returned to pre-Tx levels until 12 months post-Tx (Fig. 4a and Supplementary Fig. 4a). To exclude, that loss of Tregs was only feigned due to residual effects of basiliximab therapy, we determined CD25 expression in CD4^+^FOXP3^+^ cells, finding, that 80.95 ± 11.48% CD4^+^FOXP3^+^ cells expressed the IL-2-receptor on their cell surface (Supplementary Fig. 3).

Further we hypothesized, that over time, frequencies of activated Tregs also change in adaptation to the constant allogeneic stimulation. Therefore, we analyzed Treg activation before and after stimulation with donor MHC in a longitudinal course at following time points: pre-Tx as well as at 3, 12 and 24 months after transplantation (Fig. 4b–d). In contrast to the lower frequencies of polyspecific Tregs in the early post-Tx course, alloreactive Tregs significantly increased after kidney transplantation until three months post-Tx. From 3 to 12 months post-Tx alloactivated Treg numbers then declined and showed the lowest frequencies in the post-Tx course at 12 months after transplantation. This observation could be made for unstimulated (Fig. 4b) as well as donor-antigen stimulated PBMC (Fig. 4c and Supplementary Fig. 4b), with absolute numbers of alloactivated Tregs being higher after additional allogeneical stimulation. Notably, at the time point of 3 months with the highest frequency of activated Tregs, activated Tregs made up over 40% of the Treg niche in transplant recipients (Fig. 4c and Supplementary Fig. 4c).

### Correlation with clinical outcome

Next, we addressed the question, if alloreactive Treg frequencies are correlated with the clinical course. As at 3 months after transplantation we found the highest frequencies of activated Tregs, we decided on this time point for correlations with clinical course and compared unstimulated Tregs with Tregs after stimulation with donor-antigen and 3^rd^ party-antigen. Two patient samples could not be subjected to activation with 3^rd^ party antigen due to limited cell material.

**Figure 5:**
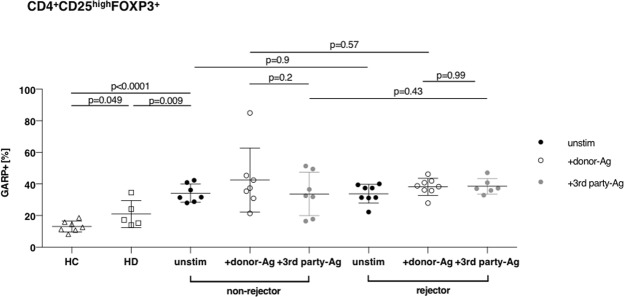
Frequency of alloreactive Tregs does not predict allograft tolerance Frequency of activated Tregs at time point 3 months of non-rejectors and rejectors are plotted in this panel. No significant differences have been observed when comparing frequency of activated Tregs in unstimulated or allogeneically (against donor and 3^rd^ party) activated Tregs in the respective groups. Data are presented as mean ± SD.

**Figure 6:**
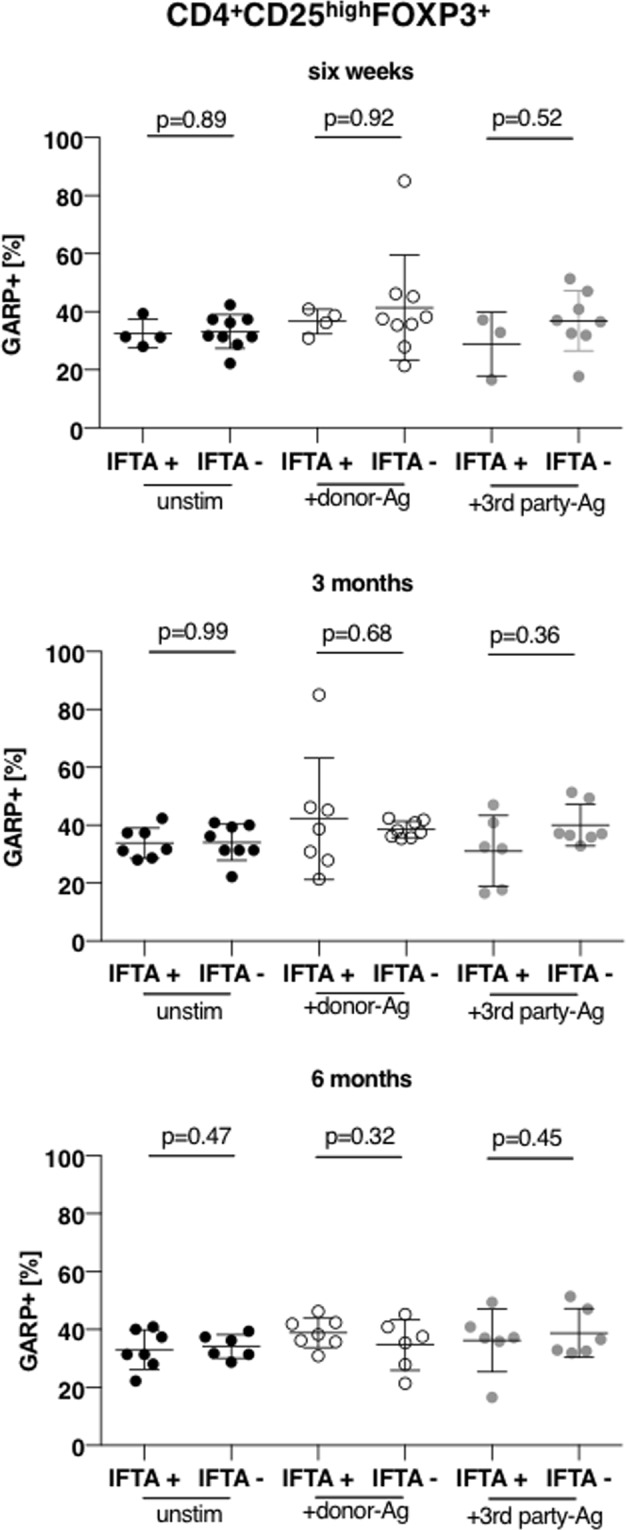
Frequency of alloreactive Tregs does not correlate with IFTA. Frequency of activated Tregs at time point 3 months were correlated with the occurrence of IFTA at six weeks, 3 months or 6 months. No significant differences have been observed when comparing frequency of activated Tregs in unstimulated or allogeneically (against donor and 3^rd^ party) activated Tregs at any time point of the occurrence of IFTA. Data are presented as mean ± SD.

**Figure 7:**
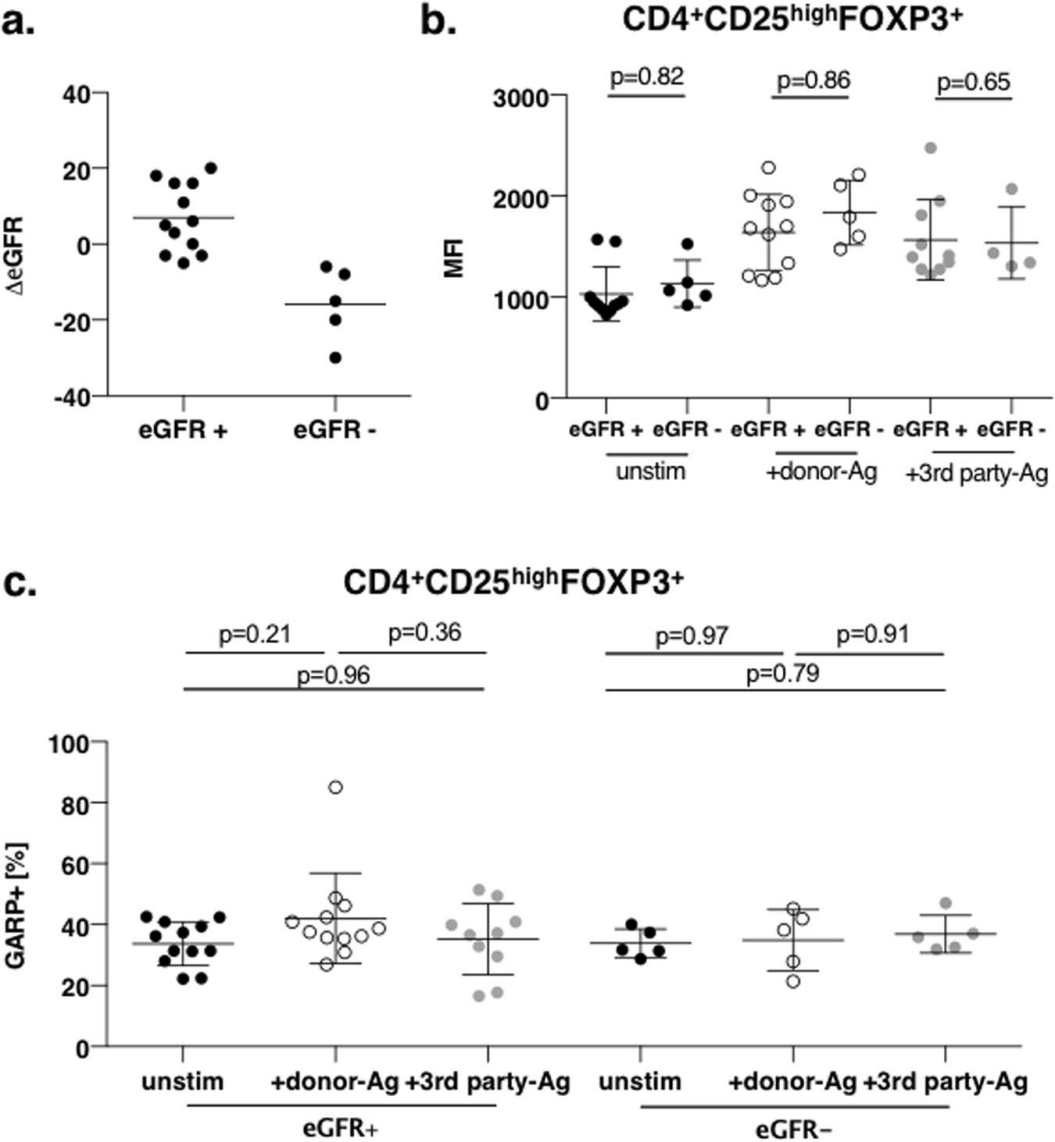
Frequency of alloreactive Tregs does not correlate with allograft function. (**a**) Overall benign clinical course of renal graft function during the observation period of 24 months is depicted. Of 17 patients, 12 patients showed a stable or improving graft function. (**b**,**c)** Frequency of activated Tregs at time point 3 months of patients with stable and worsening graft function are plotted in this panel. No significant differences have been observed when comparing frequency of activated Tregs in unstimulated or allogeneically (against donor and 3^rd^ party) activated Tregs in the respective groups. Data are presented as mean ± SD.

The classification of the patients in rejectors (n = 8) and non-rejectors (n = 7) was based on histological diagnosis of the renal allograft as described above. Frequencies of activated Tregs were distributed equally between both groups and did not correlate with ACR (Fig. 5 and Supplementary Fig. S5a). As the most common cause of renal transplant failure is interstitial fibrosis and tubular atrophy (IFTA)^34^ we correlated Tregs frequencies with IFTA at different time points (6 weeks, 3 months and 6 months, Fig. 6 and Supplementary Fig. S5b) finding no differences in activated Treg frequencies at any time point of occurrence of IFTA. Further, we questioned if Treg numbers correlate with graft function. Therefore, we determined the development of the graft function in the first 24 months after kidney transplantation by calculating the delta eGFR (estimated glomerular filtration rate, ΔeGFR = eGFR at 24 months – eGFR at 3 months). A ΔeGFR less than −5 ml/min was defined as stable or improving graft function (eGFR+), a ΔeGFR more than −5 ml/min over the time course of 24 months post-Tx was defined as worsening graft function (eGFR-). Overall, the present cohort of living donor renal grafts showed a very good post-Tx course. 70% (12/17) of patients maintained at stable or even improving graft function (Fig. 7a) and we did not detect differences in activated Tregs frequencies in patients with stable or worsening graft function (Fig. 7b,c and Supplementary Fig. S5b).

## Discussion

Approximately 3–5% of the whole T cell repertoire are Tregs^35,36^. Whereas up to 10% of the whole T cell repertoire is considered to be alloreactive^37–39^ precursor frequencies of alloreactive Tregs within the Treg niche have not been evaluated extensively due to the lack of appropriate markers for the direct identification of alloreactive Tregs. Previously, GARP has been described as surface marker expressed after polyspecific Treg activation^23,40^. In addition, we could recently show, that GARP is also a suitable marker for the identification of alloreactive Tregs^6^. Before, frequency of allospecific Tregs in healthy individuals has been estimated to 1–5% of all Tregs by means of limiting-dilutions analysis or INFγ-ELISPOT^41,42^. Our data show, that previous studies underestimated frequencies of alloreactive Tregs in healthy subjects by far, as we identified an average of 25% of all CD4^+^CD25^high^FOXP3^+^ Tregs to express GARP after allogeneic stimulation. Moreover, healthy individuals showed a marked variation in Treg alloreactivity depending on the MHC-stimulus applied.

We observed higher frequencies of polyspecific Tregs in patients with ESRD. In addition, ESRD patients also showed a significant increase of activated Tregs. The underlying reasons maybe the uremic state or hemodialysis treatment with extracorporal cell activation, but in fact remain unclear to this point. Several studies have been performed questioning frequency and function of Tregs in patients with ESRD. So far, results have been inconsistent: Increased, unaltered or decreased Treg frequencies have been reported^25–29^. In contrast to our study, all previous studies analyzed only polyspecific Tregs regardless of their activation status. None of the studies investigated the activation status of the Tregs.

Bluestone as well as others have reported that after kidney transplantation Tregs decrease temporarily as a result of induction therapy with anti-IL2R-antibody basiliximab, with a return of expression of CD25 90 days after treatment^30–33^. We can confirm these observations in our cohort, as we found a significant drop in total Treg frequency at month 3 which recovered until month 12. In order to outrule, that the observed Treg loss was only feigned due to residual modulating effects after induction therapy with the anti-IL-2 receptor blocking antibody basiliximab, we determined the expression of CD25 on CD4^+^FOXP3^+^ T cells. As almost all CD4^+^FOXP3^+^ cells (80.95 ± 11.48%) expressed CD25 on their cell surface, we could confirm, that Treg loss at 3 months after Tx is a real phenomenon (Supplementary Fig. S3).

Yet, even though total Treg numbers decreased in the first months after transplantation when immunosuppression levels are highest, we have evidence, that Treg activation is not affected by the immunosuppression as we see an expansion of activated Tregs within the Treg niche with a maximum peak at month 3 when almost 40% of all Tregs displayed an activated phenotype even before exposed to allogeneic stimulation *in vitro*. Furthermore, we hypothesize, that part of the activated Tregs are alloreactive, as allogeneic stimulation resulted in increased GARP expression in most of the samples with significant increase at the time points pre-Tx and at 12 months post-Tx. The high *ex-vivo* activation rate with a lack of GARP downregulation after prolonged resting could have been due to a strong and chronic *in vivo* alloimmune response, therefore we did not exclude these samples from the study. Yet we acknowledge, that based on our data conclusions on the specificity of Tregs, particularly alloreactivity cannot be drawn with absolute certainty. Nonetheless, independent from specificity, increased activation levels of Tregs after renal transplantation remain without question.

By trend, Tregs of non-rejectors seem to possess more donor-directed alloreactivity against third party-Ag than Tregs of rejectors, but this observation did not reach significance, maybe due to small sample number. On the other hand, it has been postulated, that CD4^+^CD25^+^ T cells are not the main mechanism of donor-reactive hyporesponsiveness in renal transplant recipients^20^. Yet these data were obtained by comparing T cell proliferation in mixed lymphocyte reactions (MLRs) before and after depletion of CD4^+^CD25^+^ T cells. As we have previously shown, polyclonal CD4^+^CD25^high^ Tregs have the weakest immunosuppressive capacity when compared to Treg subsets defined by additional markers as CD127, CD137 or GARP and LAP(latency associated protein)^6^.

Even though the general scientific opinion does not question the importance of Tregs in promoting an immunosuppressive immune response human studies on the direct impact of Tregs on the outcome of renal allografts have been controversial up to the present day.

In mostly biopsy studies, it has been postulated, that although elevated FOXP3 levels could be detected in renal allografts in ACR, this phenomenon did not correlate with graft outcome^11–13^. Ashon-Chess *et al*. neither found different FOXP3 levels in grafts nor in the peripheral blood between rejectors and non-rejectors^18^. On the contrary, Bestard *et al*. reported, that in subclinical cellular rejection FOXP3 levels were positively correlated with a favorable long-term graft outcome, even in the occurrence of IFTA^14,15^ and two other groups linked FOXP3 levels in renal borderline changes with a better graft survival^16,17^. Furthermore, Muthukumar *et al*. reported that urinary FOXP3 mRNA levels were increased in patients with ACR and that FOXP3 levels not only predicted response to anti-rejection therapy but also identified patients at risk for graft failure within 6 months after the incident episode of acute rejection^43^.

In our cohort, independent from the clinical outcome, frequency of activated Tregs increased in the first 3 months after transplantation and then declined to pre-Tx baseline levels within one year after transplantation and stayed stable in the observation period of 24 months. Although we neither found correlations between the extent of activation of circulating activated Tregs and graft acceptance, nor observed an effect on graft function or IFTA, there is evidence that Tregs can be linked to clinical outcome: Vondran *et al*. previously reported that renal allograft recipients with lower pretransplant Tregs frequencies were at higher risk for ACR^44^. Furthermore, Salman *et al*. just recently showed, that Tregs frequencies determined as early as 3 weeks after transplantation predict development of bronchiolitis obliterans in a lung recipient cohort^45^. And in a recent study conducted by Leventhal *et al*., increase of Treg frequency after kidney transplantation in patients treated with adoptive transfer of donor hematopoetic stem cells was associated with tolerance of the renal graft^46,47^.

One possible reason, why we could not link Tregs number and phenotype to clinical outcome was the benign clinical course of our living donor kidney transplantation cohort, in which the majority of 70% had a very stable course with stable graft function. ACR mainly occurred in the early weeks after transplantation. Therefore, the analyzed cohort in general did not present significant differences in their clinical course, which is not surprising as living donation is known to have a better graft outcome^48^. Yet, our question of donor-directed alloreactivity was dependent on the availability of donor material, therefore we chose to perform our study in a living donor kidney transplant cohort.

In summary, we could prove for the first time that after renal transplantation alloreactive and activated Tregs increase significantly representing up to 40% of all peripheral Tregs. The results are important for current clinical trials using adoptive Treg therapy after kidney transplantation^49–52^. Adoptively transferred Tregs could be important to compensate the Treg loss at month 3, while they have to compete within the Treg niche with a large number of activated Tregs.

## Methods

### Patient characteristics

We included 17 patients, who underwent living donor kidney transplantation between 2008 and 2010 at Hannover Medical School, Germany and had been enrolled in a previous study conducted by Vondran *et al*.^44^. For the purpose of comparison of the allogeneic immune response in patients with and without acute cellular rejection, patient groups (non-rejectors and rejectors) were aimed to consist of similar numbers. Therefore, we selected 7 patients with rejection-free post-Tx course and 8 patients with at least one episode of ACR defined by biopsy according to BANFF classification^53^ in the observation period of 24 months from the initial patient cohort. Furthermore, two additional patients could be recruited for the study, but these two patients only agreed to blood donation and no kidney transplant biopsy. Patients with a history of previous organ transplantation were excluded from this study.

The 15 patients (88%), who were included in the local renal transplant control biopsy program had biopsies taken at following time points: 11 patients were biopsied at 6 weeks, 3 months and 6 months after Tx; 4 patients were biopsied only at 3 months and 6 months post-Tx. The two patients who did not participate in the control biopsy program had no biopsy done. 15 patients (88%) required hemodialysis prior to transplantation, one patient performed peritoneal dialysis, one patient received preemptive kidney transplantation. According to local standards, a triple immunosuppression consisting of a calcineurin inhibitor (CNI) (17/17, tacrolimus (Tac) n = 15, cyclosporin A (CyA) n = 2), mycophenolate mofetil (MMF) (16/17) and corticosteroids (17/17) was administered after transplantation. Trough levels for tacrolimus (15 patients) were 8 µg/l (range 6–10 µg/l) at 3 months post-Tx, 6 µg/l at 1 year post-Tx (range 5–9 µg/l) and 6 µg/l (range 4–9 µg/l) 2 years post-Tx. Trough levels for cyclosporine A (2 patients) were 60 and 76 µg/l, respectively, in the first two years after transplantation. One patient was treated with mTOR (mammalian target of rapamycin) inhibitor sirolimus (trough level 6 µg/l) instead of MMF, another patient was switched from MMF to azathioprine after 4 months. 15 (88%) patients received an induction therapy with either anti-CD25 antibody basiliximab (14/15) or anti-CD20 antibody rituximab (1/15, ABO incompatible Tx), 2 patients received no induction therapy. Graft function was assessed using the Modification of Diet in Renal Disease (MDRD) Study serum creatinine-based equation to calculate estimated glomerular filtration rates (eGFR). For the purpose of better comparability, we calculated the ΔeGFR from 24 months (end of observation period) and 3 months after transplantation for each patient (Table 1).

**Table 1:** Patient characteristics.

Sex	male	11
	female	6
Age at Tx (years)		44.6 ± 13.4
Allograft tolerance	non-rejector	7
	rejection	8
	no biopsy	2
Timepoint of ACR	before day 10	5
	day 40 and day 90**	1
	day 124	1
	day 544	1
Biopsies obtained	6 weeks	13
	3 months	15
	6 months	13
Dialysis prior to Tx	hemodialysis	15
	peritoneal dialysis	1
	preemptive Tx	1
Mean duration of dialysis (months)		26.8 ± 22.9
Immunosuppression	Tac/MMF/Steroids	14
	CyA/MMF/Steroids	2
	Tac/Aza/Steroids	1*
	Tac/Sirolimus/Steroids	1
Induction therapy	basiliximab	14
	rituximab	1
	no induction	2
Mean eGFR [ml/min]	3 months	52.9 ± 16
	24 months	55.7 ± 16.8
delta eGFR (24 months–3 months) (non-rejector/rejector/no biopsy)	>10 ml/min	5 (2/2/1)
	>−5/<5 ml/min	7 (3/3/1)
	<−5 ml/min	5 (3/2)

*One patient was switched from MMF to Aza after 4 months.

**Same patient had 2 episodes of ACR within 24 months.

Informed consent to participate in this study was obtained from all patients and their appropriate donors. No blood samples or biopsy was procured from prisoners. Blood samples and kidney biopsies were procured by the Department of General, Visceral and Transplantation Surgery and the Department of Nephrology of Hannover Medical School, Germany. This study was approved by the ethics committee of Hannover Medical School (approval number: 894–2010). The authors declare that this study adhered to the Declaration of Helsinki and to the Declaration of Istanbul.

### Blood samples

Heparinized blood samples of all 17 patients were collected at three months post-Tx. Out of these 17 patients, ten patients also donated blood samples for longitudinal analysis before renal transplantation, as well as 12 and 24 months afterwards. Of these, six patients had a rejection free post-Tx course, two patients experienced episodes of ACR (one patient at day 7 and the other patient at day 40 and 90) and the remaning two patients had no biopsies done.

For analysis of donor-specific Treg responses blood samples of all 17 kidney donors were obtained. Furthermore, donor-specific T-cell responses of kidney recipients were compared with responses to third party antigen. As described before, suitable healthy third party controls were chosen for their identical number of mismatches with the recipients regarding the HLA-A, -B and -DR loci as in the donor/recipient constellation^44^ (Supplementary Table S1). HLA-typing was conducted according to ASHI standards. For controls, 7 healthy volunteers (group HC) and 5 patients on chronic hemodialysis (group HD) donated heparinized blood samples.

Peripheral blood mononuclear cells (PBMC) were obtained by ficoll density gradient centrifugation and stored in liquid nitrogen in RPMI1640/ 20% fetal calf serum/10% DMSO until further use.

### Mixed lymphocyte reaction

Frozen PBMCs were thawed and rested for 5 days in RPMI1640 supplemented with 1% penicillin/streptomycin, 10% FCS, 50 mM b-Mercaptoethanol and 0.1 M HEPES in humidified incubators at 37 °C and 5% CO_2_. For mixed lymphocyte reactions (MLR) stimulator PBMC (from donor and third party, respectively) were labelled with 5 mM CFSE (37 °C for 10 minutes) for later distinct discrimination from responder recipient’s PBMC in flow cytometry before they were lethally irradiated with 30 Gy. MLRs were performed in a ratio of 3:1 (stimulator: recipient) in the presence of low-dose IL-2 (20 U/ml) for 24 hours. Therefore, 3 × 10^5^ recipient-PBMC were co-cultured with 9 × 10^5^ irradiated and CFSE-labelled donor-PBMCs in 96-U bottom culture plates in 200 µl medium.

### Flow cytometry

For the detection of allogeneically activated Tregs multicolor flow cytometry was performed using a combination of following monoclonal antibodies: anti-human CD4 Brilliant Violet 421 (clone: RPA-T4, biolegend), anti-human CD25 PE-Cy7 (clone: M-A251, biolegend), anti-human GARP PE (clone: ME-9FI, biolegend). Intracellular staining of FOXP3 APC (ebioscience, clone: PCH101) was performed according to manufacturer’s instructions. Subsequent flow cytometry was performed on a LSR II (BD Biosciences). Stimulator PBMCs were identified by CFSE labelling and excluded from the analysis. Tregs were identified as CD4^+^CD25^high^FOXP3^+^, activated Tregs were identified by their co-expression of GARP. Frequency of activated Tregs was calculated as ratio of CD4^+^CD25^high^FOXP3^+^GARP^+^ to CD4^+^CD25^high^FOXP3^+^ (Supplementary Fig. 1).

### Flow cytometric analysis

Flow cytometric analysis was performed using FlowJo Software, Tree Star, Inc.

### Statistical analysis

Statistical analyses were performed using GraphPad PRISM version 6.0 for MacOSX, GraphPad Software, San Diego California USA. Significant differences were calculated based on student t-test or ONE-way ANOVA when indicated. A p-value < 0.05 was considered as statistically significant.

## Supplementary information

Transient increase of activated regulatory T cells early after kidney transplantation

## Data Availability

All data generated or analysed during this study are included in this published article (and its Supplementary Information files).
