# Supplementary material for: Transient increase of activated regulatory T cells early after kidney transplantation
Source: Sci Rep. 2019 Jan 31;9:1021. doi: 10.1038/s41598-018-37218-x (PMC6355855; doi:10.1038/s41598-018-37218-x)
Supplement: Supplementary file 1 — Transient increase of activated regulatory T cells early after kidney transplantation [file 41598_2018_37218_MOESM1_ESM.pdf]

Transient increase of activated regulatory T cells early after kidney transplantation

Young-Seon Mederacke[1#], Florian W. Vondran[2#], Sonja Kollrich[2], Elvira Schulde[1], Roland Schmitt[3,], Michael P. Manns[1], Jürgen Klempnauer[2], Reinhard Schwinzer[2], Fatih Noyan[1,4+], Elmar Jaeckel[1,4+*]

[1] Dept. of Gastroenterology, Hepatology & Endocrinology, Hannover Medical School, Hannover, Germany

[2]Dept. of General, Visceral and Transplantation Surgery, Hannover Medical School, Hannover Germany

[3]Dept. of Nephrology, Hannover Medical School, Hannover, Germany

[4] Integrated Research and Treatment Center, Transplantation (IFB-Tx), Hannover Medical School, Hannover, Germany

[#]contributed equally

[+]contributed equally

Correspondence should be addressed to:

Elmar Jaeckel, MD

Dept. of Gastroenterology, Hepatology & Endocrinology

Hannover Medical School, Carl-Neuberg-Str. 1, D-30625 Hannover, Germany

Phone +49-511-532 9513

Fax +49-511-532 6998

E-mail: Jaeckel.Elmar@mh-hannover.de

Supplementary figure S1

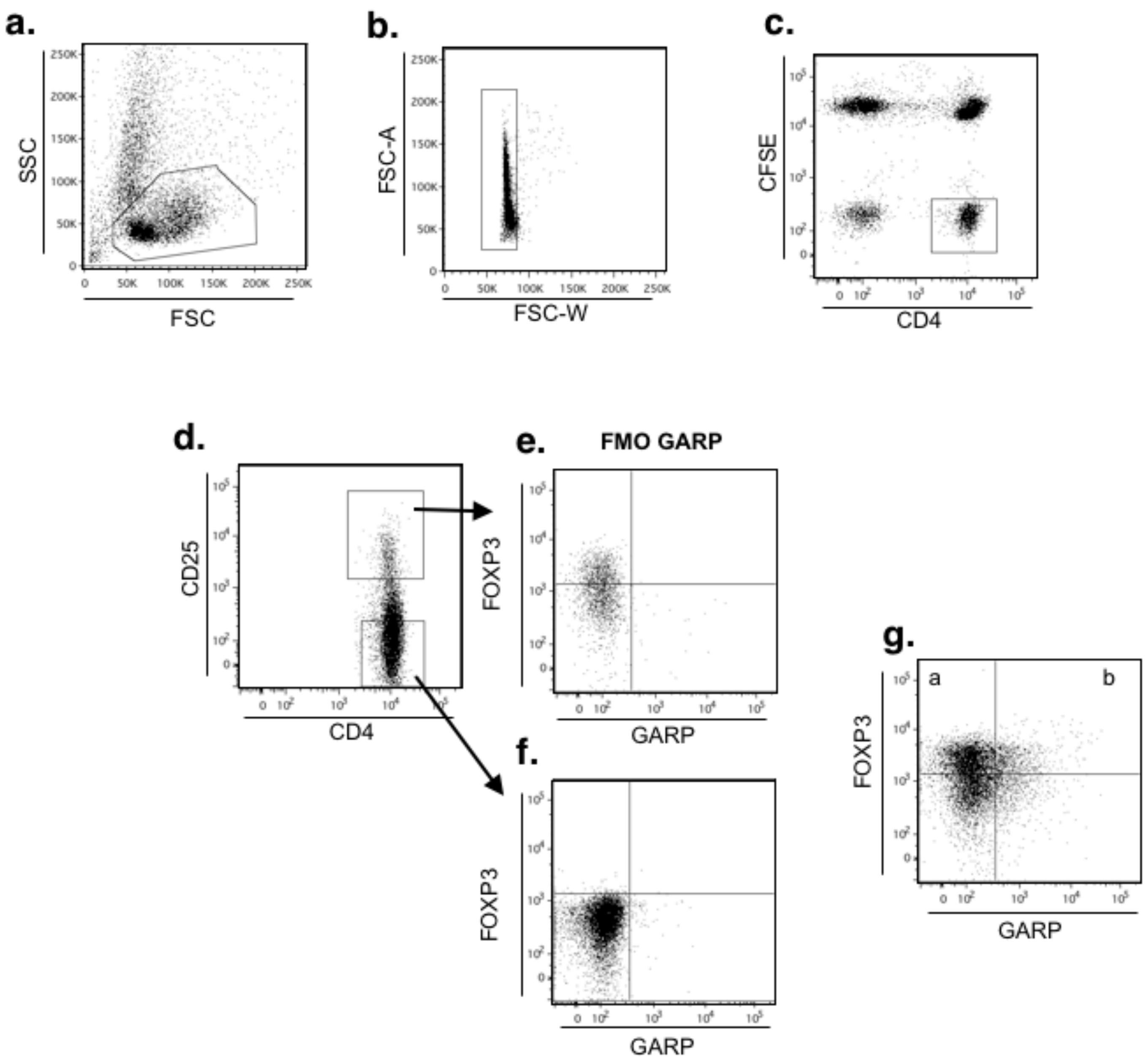

Supplementary figure S1: Gating strategy for activated Tregs

Gating was performed as follows: Gating for lymphocytes **(a)** with subsequent gating for single cells to eliminate doublets **(b)**. Recipient's CD4^{+} T cells were separated from CFSE^{+} donor cells **(c)**. Pregating on CD4^{+}CD25^{high} cells **(d)**. Gate for FOXP3^{+} cells was set using CD25^{-} cells as internal control for FOXP3 **(e)**. For the determination of GARP^{+} cells, a FMO staining for GARP was applied **(f)**. Frequency of activated GARP^{+} Tregs was calculated by the ratio of quadrant b/quadrant a+b **(g)**.

Supplementary figure S2

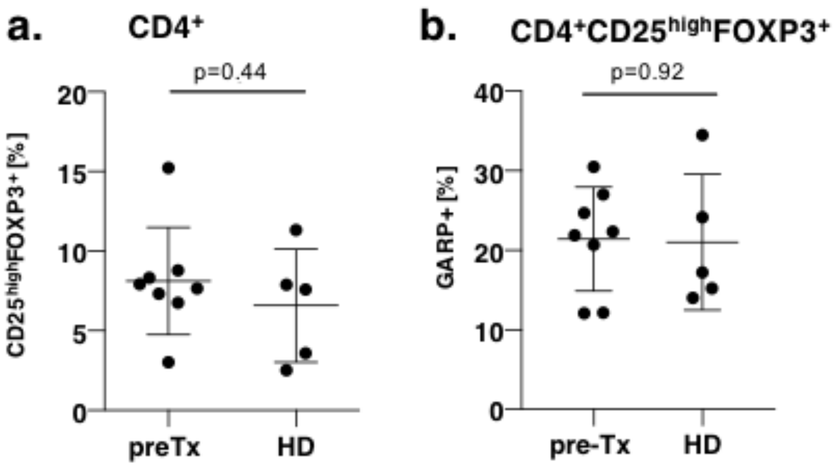

Supplementary figure S2: Frequency of polyspecific and activated Tregs in patients with ESRD

Frequencies of $CD4^{+}CD25^{high}FOXP3^{+}$ polyspecific regulatory T cells **(a)** and endogenously activated $CD4^{+}CD25^{high}FOXP3^{+}GARP^{+}$ regulatory T cells **(b)** was assessed in unstimulated PBMC of 8 patients from the study cohort pre-Tx and 5 patients who were on chronic hemodialysis. No statistically significant difference was detected, therefore all patients were combined as ESRD group.

Supplemetary figure S3

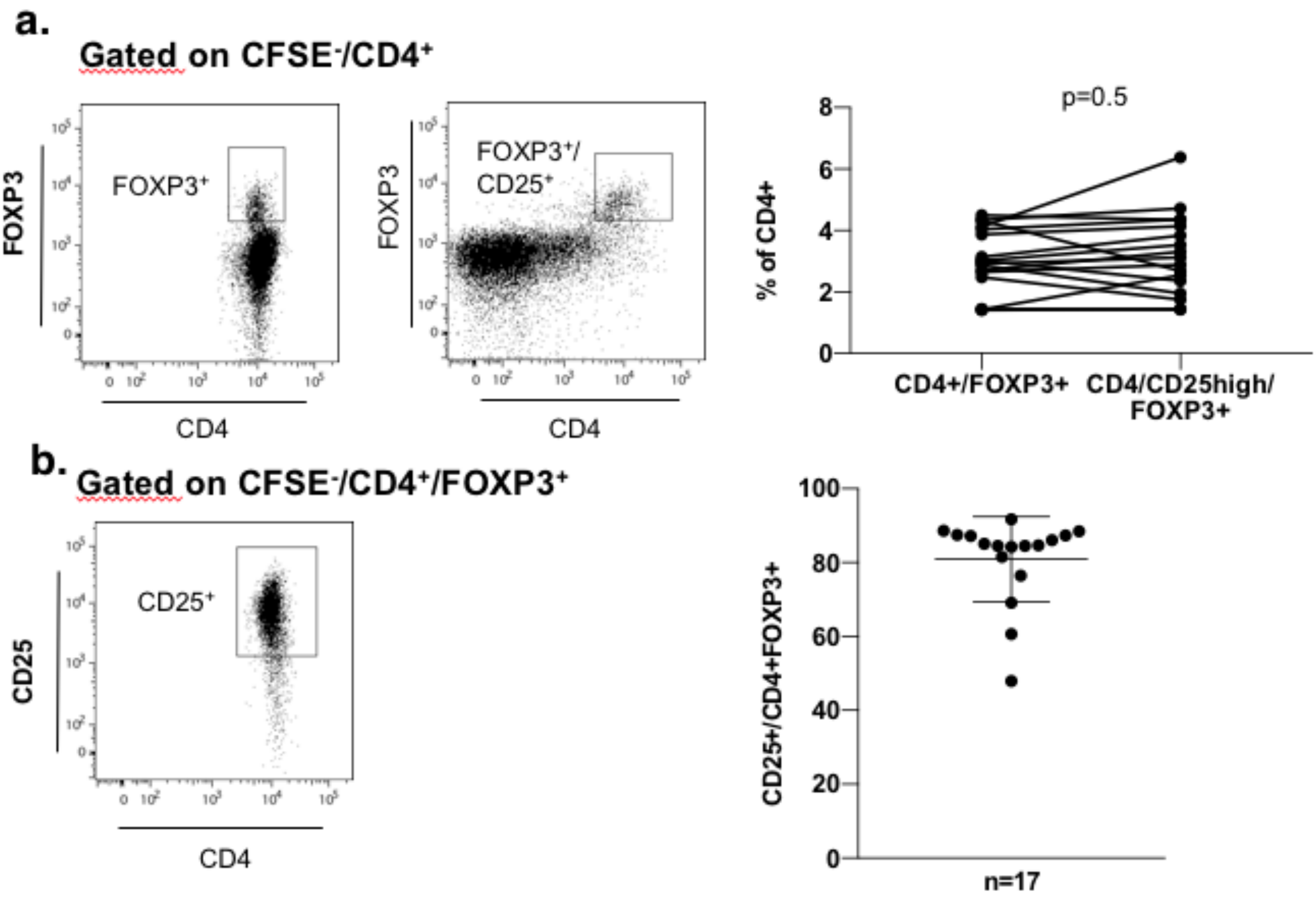

Supplementary figure S3: Determination of Tregs at time point 3 months

To outrule bias regarding Treg frequency at the time point of three months introduced by CD25 modulation due to induction therapy with anti-CD25 antibody basiliximab, two different gating strategies were applied for the identification of Tregs.

a. $CD4^+$ Tregs were identified by their high expression of CD25 and FOXP3, or by FOXP3 expression only. Identification of Tregs using these two gating strategies resulted in comparable numbers of Tregs (3.08 ± 1.02% vs 3.23 ± 1.32%, p=0.5).

b. Expression of CD25 on $CD4^+FOXP3^+$ cells was confirmed on 80.95 ± 11.48% of all $CD4^+FOXP3^+$cells.

Supplementary figure S4

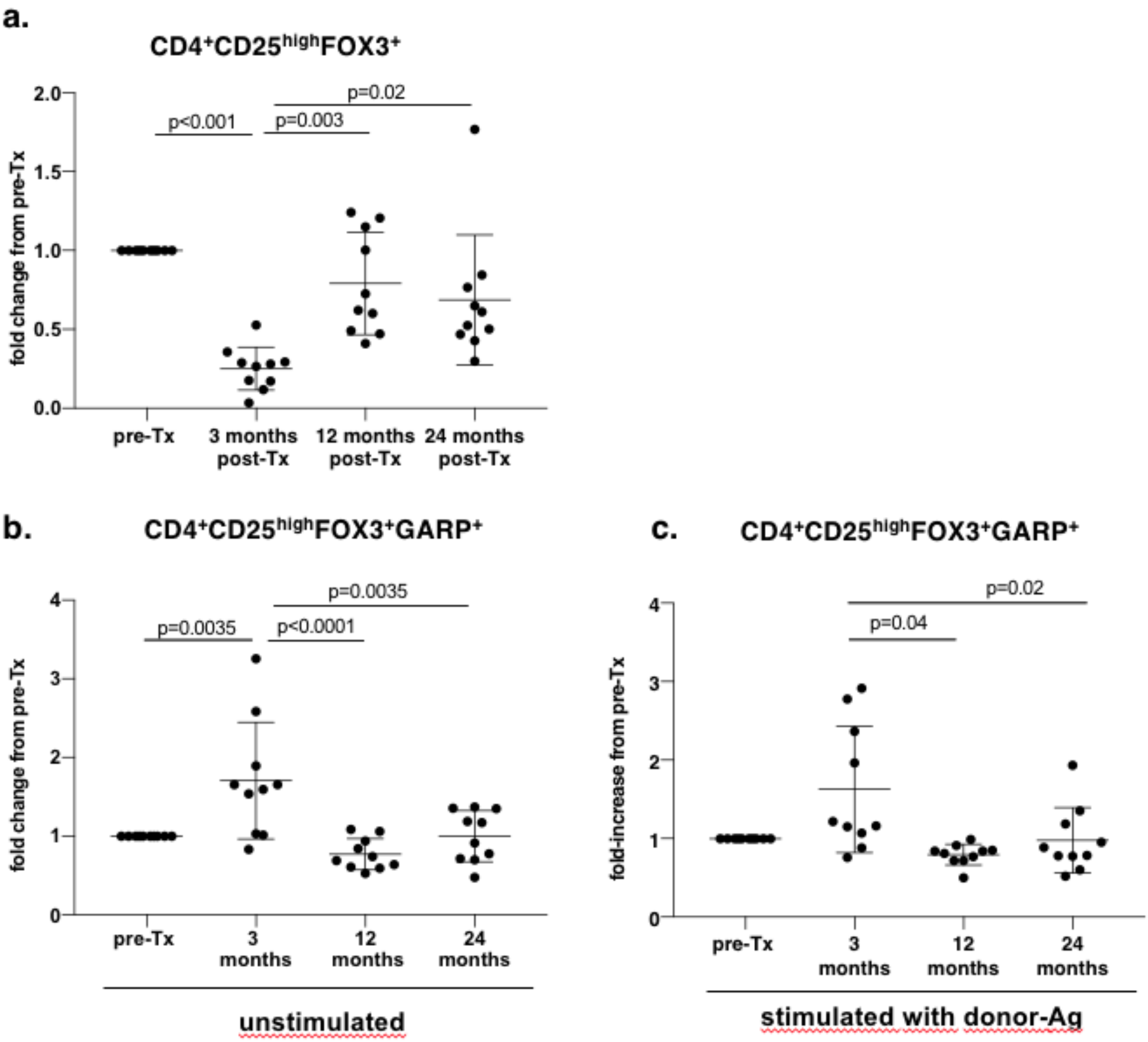

Supplementary figure S4: Fold change of the Treg frequency percentages compared with pre-Tx values in the longitudinal course after kidney Tx.

a. Frequency of polyspecific Tregs drop by 4fold in the first three months after kidney transplantation when compared to pre-Tx values. In the further longitudinal course Treg frequency increases again until 12 months post-Tx and stays stable until 24 months post-Tx. Pre-Tx values were normalized to 1. Data are presented as mean ± SD.

b + c. Frequency of activated Tregs in unstimulated **(b)** and with donor-Ag stimulated Tregs **(c)** in the longitudinal course after Tx. Pre-Tx values were normalized to 1. Data are presented as mean ± SD.

Supplementary figure S5

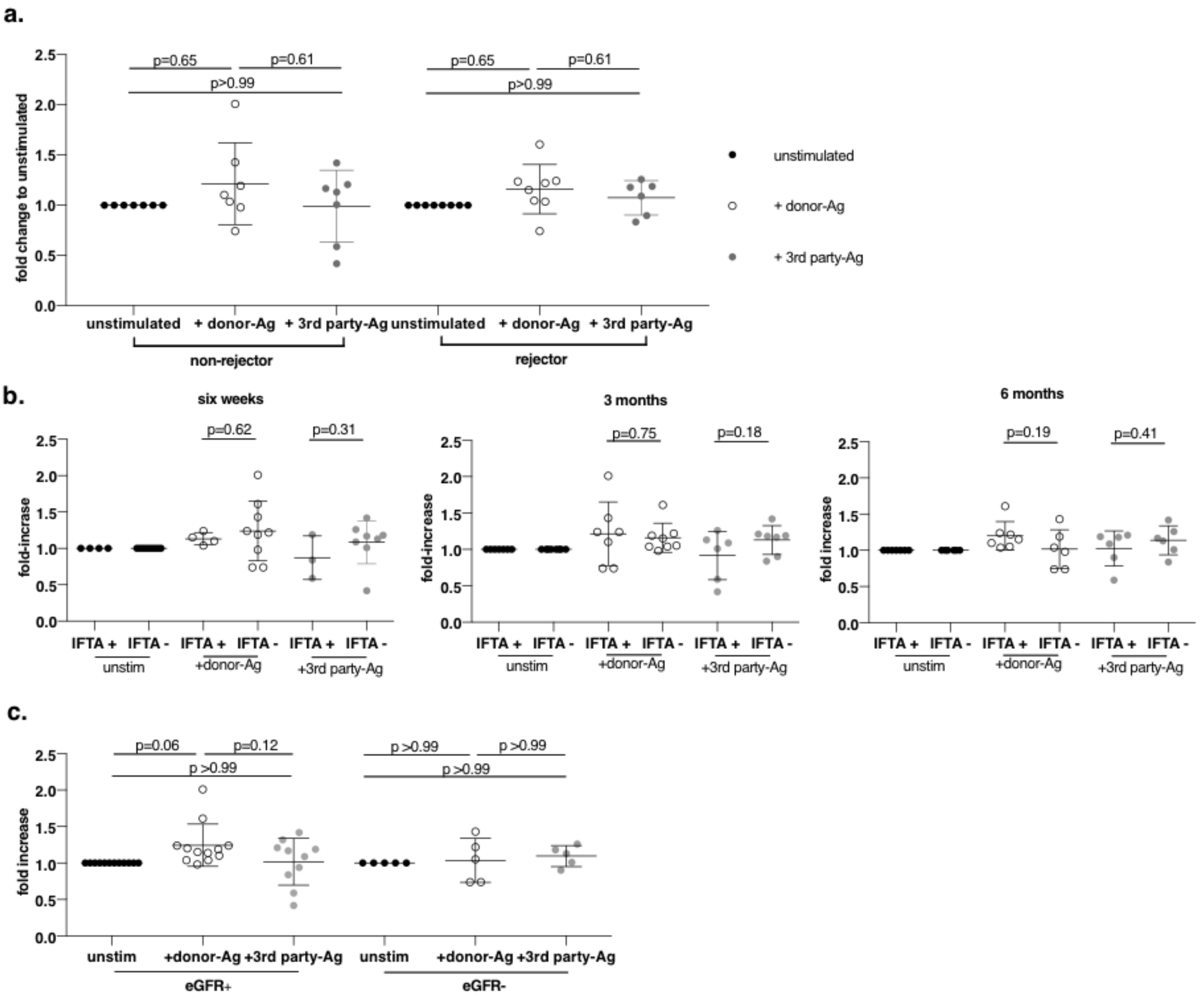

Supplementary figure S5: Treg frequency changes do not predict allograft tolerance, graft function or the occurrence of IFTA

Treg frequencies after stimulation with allogeneic antigen (donor or 3rd party) were normalized to their respective unstimulated sample at the time point of 3 months after Tx. At this timepoint, no significant activation after stimulation with alloantigen is detectable in neither rejectors nor non-rejectors **(a)**. In addition, Treg frequencies do not predict the occurrence of IFTA **(b)** nor the development of the renal allograft function **(c).** Frequencies of unstimulated Tregs were normalized to 1. Data are presented as mean ± SD.

Supplementary Table S1: HLA-matching and 3rd party controls

	Recipient			Donor			3rd party			Mismatch
#	HLA-A	HLA-B	HLA-DR	HLA-A	HLA-B	HLA-DR	HLA-A	HLA-B	HLA-DR	(R/D ; R/3rd)
01	***01, 26***	***38, 37***	***11, 14***	***03, 30***	***13, 07***	***13, 15***	***03, 68***	***40, 57***	***03, 04***	***2 – 2 – 2***
02	01, 01	08, 37	03, 10	02, 02	07, 55	04, 15	24, 24	07, 39	01, 11	1 – 2 – 2
03	***11, 69***	***35, 35***	***01, 04***	***02, 02***	***44, 55***	***04, 04***	***01, 68***	***08, 15***	***01, 04***	***1 – 2 – 0***
04	11, 30	13, 55	14, 07	02, 11	51, 55	08, 14	02, 02	18, 18	11, 11	1 – 1 – 1
05	***24, 31***	***44, 62***	***16, 04***	***01, 23***	***44, 73***	***13, 07***	***02, 24***	***44, 50***	***07, 11***	***1 – 1 – 2***
06	24, 31	07, 62	15, 11	03, 11	44, 50	07, 08	01, 02	08, 18	01, 03	2 – 2 – 2
07	***01, 01***	***08, 08***	***03, 03***	***01, 11***	***08, 44***	***03, 11***	***01, 02***	***08, 38***	***03, 13***	***1 – 1 – 1***
08	26, 32	08, 27	15, 03	03, 26	15, 27	13, 15	02, 02	18, 18	11, 11	1 – 1 – 1
09	***26, 69***	***51, 38***	***04, 10***	***24, 26***	***18, 38***	***04, 11***	***02, 26***	***27, 38***	***04, 12***	***1 – 1 – 1***
10	02, 11	51, 35	11, 14	68, 68	14, 44	04, 13	02, 31	07, 39	04, 11	1 – 1 – 2
11	***01, 11***	***35, 37***	***01, 04***	***03, 26***	***07, 07***	***11, 15***	***02, 29***	***44, 44***	***07, 11***	***2 – 1 – 2***
12	02, 24	51, 18	15, 11	01, 02	35, 51	01, 11	02, 11	18, 40	04, 11	1 – 1 – 1
13	***02, 03***	***07, 56***	***04, 08***	***02, 02***	***56, 57***	***07, 08***	***02, 02***	***40, 40***	***04, 13***	***0 – 1 – 1***
14	02, 02	44, 37	04, 10	01, 02	08, 37	03, 10	02, 24	07, 44	04, 15	1 – 1 – 1
15	***03, 24***	***15, 38***	***15, 13***	***01, 26***	***08, 27***	***01, 03***	***02, 11***	***18, 40***	***04, 11***	***2 – 2 – 2***
16	01, 30	08, 13	03, 07	24, 26	18, 44	13, 15	02, 11	18, 40	04, 11	2 – 2 – 2
17	***02, 03***	***35, 40***	***01, 13***	***02, 31***	***07, 27***	***01, 15***	***01, 02***	***08, 18***	***01, 03***	***1 – 2 – 1***

HLA = human leukocyte antigen; R/D = Recipient vs. Donor, R/3rd = Recipient vs. 3rd party.
